# The challenges and opportunities of social connection when hearing derogatory and threatening voices: A thematic analysis with patients experiencing psychosis

**DOI:** 10.1111/papt.12303

**Published:** 2020-10-30

**Authors:** Bryony Sheaves, Louise Johns, Emma Černis, Laura Griffith, The McPin Hearing Voices Lived Experience Advisory Panel, Daniel Freeman

**Affiliations:** ^1^ Department of Psychiatry University of Oxford UK; ^2^ Oxford Health NHS Foundation Trust UK; ^3^ Public Health England London UK; ^4^ The McPin Foundation London UK

**Keywords:** auditory hallucinations, loneliness, psychosis, social connection, social isolation, voice hearing

## Abstract

**Objectives:**

Relationships with other people are important determinants of the course of psychosis, yet social isolation is common. This study sought to learn about the patient experience of being around other people when hearing derogatory and threatening voices (DTVs).

**Design:**

A qualitative interview study.

**Methods:**

Fifteen participants with experience of hearing DTVs in the context of non‐affective psychosis were recruited from NHS services. Data were obtained by semi‐structured interviews and analysed using thematic analysis.

**Results:**

Three themes were identified: (1) reasons why interacting with people is difficult when hearing DTVs; (2) the relationship between social connection and DTVs; and (3) factors which enable voice hearers to connect with others. A further ten sub‐themes are outlined as reasons why hearing DTVs led to lower social connection, including difficulties during conversations (e.g., *the* *concentration required is hard*), negative expectations of interactions (e.g., *fearing negative judgement from others*), and difficulties sharing experiences of voices (e.g., *people will be hurt or upset if I tell them about the voices*). Isolation was a common response to hearing DTVs but also a time of vulnerability for hearing voices. Managing the challenges of interacting with people led to some improvements in DTVs.

**Conclusions:**

There are understandable reasons why hearing DTVs leads to lower social connection. Yet isolating oneself can also be a time of vulnerability for DTVs. Social connection might be one vehicle for disengaging from and disputing derogatory and threatening voice content. The effect on voice hearing of social recovery interventions warrants further investigation.

**Practitioner points:**

- Participants shared 10 reasons why being around people is challenging when hearing derogatory and threatening voices. These typically affected both daily social experiences and contact with clinicians.
- Common initial responses to hearing DTVs were to reduce contact with people, experience difficulties connecting during conversations and to avoid sharing the experience of voice hearing.
- However, social isolation was a time of vulnerability to DTVs, and hence, increasing social connection might be a target for interventions.
- A range of factors enabled voice hearers to manage social situations, for example: the fostering of trust, self‐acceptance, learning when it is better to stay at home, and developing a narrative to explain voice hearing to others.
- Addressing the barriers to connecting with others might have an important role in personal recovery from voice hearing.

## Background

*“the worst thing I did was when I was 16 I didn’t tell anyone. And then I ended up like having a complete breakdown when I was 18 and I think that could have been averted if I had actually reached out to someone”*. (V7)

Feelings of loneliness and social isolation are common in psychosis (Meltzer *et al*., 2013; Michalska Da Rocha, Rhodes, Vasilopoulou, & Hutton, 2018) and evident even from a first episode (Sündermann, Onwumere, Kane, Morgan, & Kuipers, 2014). Social isolation is an important issue in itself and also predicts later severity of symptoms (Norman *et al*., 2005; Salokangas, 1997) and the likelihood of a later inpatient admission (Norman *et al*., 2005). The odds of psychotic experiences persisting in the general population is halved for those who are married, compared with single people (DeVylder, Lehmann, & Chen, 2015), and being single whilst living in a neighbourhood with fewer single people increases the incidence of schizophrenia, likely due to increased isolation (Van Os, Driessen, Gunther, & Delespaul, 2000). Relationships with other people are clearly important determinants of the course of psychosis, yet understanding the reasons why patients become isolated and ways in which they might overcome isolation has received surprisingly little academic focus.

### Reasons why hearing derogatory and threatening voices might lead to lower social connection

The challenges that social interactions bring to people who hear distressing voices are likely to be different to those of other experientially distinct psychotic experiences (e.g., grandiose delusions). They may also differ according to voice presentation (companionate or commanding vs. threatening voices). This paper therefore focuses on the experience of being around other people in the context of one specific presentation: derogatory and threatening voices (DTVs).

There are several candidate reasons why DTVs might reduce the quality and availability of relationships. The stress of stigma (Xu *et al*., 2016) and what voices actually say (e.g., that the patient is in danger) can lead voice hearers to isolate themselves. Being distracted by voices can decrease the quality of interactions, and patient aggression towards others can result in physical distancing in order to maintain safety (Onwumere, Parkyn, Learmonth, & Kuipers, 2019). Conversation topics may also be important. Hearing DTVs may be the most salient topic of conversation for a voice hearer. However, there are several barriers to disclosing the experience of voice hearing including concerns about upsetting others and fear of stigma and resultant shame (Bogen‐Johnston, de Visser, Strauss, Berry, & Hayward, 2017; Mawson, Berry, Murray, & Hayward, 2011; Watkins, Gupta, & Sanderson, 2019). This reduces the common ground for conversation topics.

### Aim

The current study sought to build on this literature by learning from patients’ experiences of being around people whilst hearing DTVs.

## Method

### Participants

The qualitative interview data were obtained within a study (published separately) which had a primary question: ‘why do people listen to and believe DTVs?’ (Sheaves *et al*., 2020). Theoretical sampling was used to recruit participants for the purpose of grounded theory analysis linked to that primary (but separate) research question. A pilot stage, early in sampling, ensured a diversity of: age, duration of hearing voices, and employment status. Subsequently, theoretical sampling and eventual theoretical saturation (Corbin & Strauss, 2015) guided recruitment for the purpose of generating the theory reported in Sheaves *et al*. (2020). Hence, recruitment was not guided by the aim of the current study.

Fifteen participants were sought from clinical teams within Oxford Health NHS Foundation Trust. BS completed a telephone screen with the referrer and participant. Inclusion criteria were as follows: daily experience (current or past) of DTVs; experience of DTVs for at least three months; and willing and able to recall and discuss their experience in detail; fluent in English; age 18‐65; willing and able to provide informed consent. Exclusion criteria were as follows: moderate‐to‐severe learning disability; voices caused by an organic syndrome (e.g., dementia, head injury); and voices occurring solely within the context of substance misuse, personality disorder, or a mood episode (depression or mania). Two participants had previously met the interviewer through participation in research trials. The remaining participants were unknown to the researcher.

The sample was predominantly single, White British, unemployed, and lived either alone or with parents (Table 1). All but one participant currently heard DTVs. The majority reported hearing voices whilst talking to other people, but found them either less frequent or more in the background of attention when compared to hearing voices when they were alone.

**Table 1:** Demographics and clinical characteristics (*N* = 15)

Demographic or clinical characteristics	Frequency
Age	
≤20	1
21–30	3
31–40	5
41–50	5
51–60	1
Ethnicity	
White British	13
Chinese	1
Black British	1
Marital status	
Single	11
Married/civil partnership	3
Divorced	1
Employment status	
Unemployed	10
Employed (part time)	2
Self‐employed	1
Student	1
Voluntary work	1
Accommodation status	
Lives alone	6
Lives with parents	6
Lives with partner/spouse	2
Supported accommodation	1
Diagnosis	
Schizophrenia	10
Schizoaffective disorder	1
Psychosis NOS	4
‘Do/did you hear voices when talking to other people? What is/was that like?’	
Yes	4
Yes, but less often than alone	3
Yes, but more in the background	3
No	3
N/A	2
Reported testing voice content by discussing with other people^a^	
Yes, successful test	4
Yes, unsuccessful test	2
No	8
N/A	1
Age of onset of voice hearing	
≤15	4
16–25	7
26–35	2
36–45	1
46–55	1
Duration of voice hearing (years)	
1–5 years	5
6–10 years	0
11–15 years	3
16–20 years	4
21–25 years	2
26–30 years	1

Note

^a^ For example, asking if other people also heard the voice or sharing voice content for the purpose of testing it.

### Procedure

The study received NHS ethical approval (ref: 18/SC/0443). LG (a qualitative methodologist), BS, DF, and LJ (clinical psychologists and academics specializing in psychosis research) and a lived experience advisory panel (LEAP) consulted on the protocol, topic guide, interview process, and emerging themes. An audio‐recorded semi‐structured interview was conducted with BS and transcribed verbatim. Interviews were one to one, lasted around 90 min, and took place at the participant’s home or NHS clinic. The researcher’s background and motivation for conducting the research was shared, and the participants’ expertise through their experience emphasized to minimize any potential power imbalance. The interview intended to generate the participants’ own detailed description of their experience rather than merely responding to closed questions. One question probed social interactions whilst hearing voices (*‘Do/did you hear voices when talking to other people? What is/was that like?’*) which was asked to all participants. Relationships with others were also raised by participants, unprompted, throughout the interview (e.g., when sharing times that voices were better or worse, or advice for other voice hearers). Further details on the interview process are in Supporting Information.

### Analysis

Thematic analysis was used to identify substantive themes across the data set. Data familiarization involved listening to the audio tape whilst reading the transcript. A broad code of ‘voices and social interactions’ was generated to capture any data related to the research question alongside codes for other interpersonal concepts derived from the data (e.g., ‘trust’ and ‘social isolation’). This initial coding took place in parallel to coding for Sheaves *et al*. (2020). BS (who also conducted the interviews) conducted all analysis, and EČ assessed the appropriateness of each code from those provided by BS. Both are clinical psychologists with experience of psychosis research. Discrepancies were discussed and a consensus agreed. NVivo 12 (QSR International Pty Ltd, 2018) supported the data coding, organization, and analysis. Initial analysis revealed reasons why hearing DTVs led to isolation and reasons why social interactions can alleviate DTVs. Preliminary analysis was discussed with the LEAP and two clinical psychologists (DF and LJ), and additional codes were added as follows: 1) Why long‐term isolation is not the best solution for voice hearing and 2) ways of managing interactions. All transcripts were subsequently recoded using this refined framework. A research diary was kept throughout the interviewing and analysis process.

## Results

Three themes were identified as being important to the experience of being around people when hearing DTVs: (1) reasons why interacting with people is difficult when hearing DTVs; (2) the relationship between social connection and DTVs; and (3) factors which enabled voice hearers to connect with others. Each theme, their corresponding sub‐themes, and supporting quotes are outlined below.

### Reasons why interacting with people is difficult whilst hearing DTVs

#### The comments of the voices reduce my trust in others

Voices reduced trust in others by making direct comments *‘the voices are telling me people are going to hurt me [..] I can’t really walk past people’* (V10). This included comments about a therapist *‘just because you have had like one meeting [..]they would be like don’t think [..] they are not going to do anything’*. (V10). Mistrust towards the voices also became generalized to everyone *‘you can’t trust people [..] it sort of reflects into my social life because I find it difficult to trust people’* (V13). A participant who heard the voice of a family friend described the impact on trusting her mum: *‘I thought that was her helping [the voice], so that changed my view on her for a while’* (V8).

#### If I talk to people about the voices, it will hurt or upset them

Many participants felt unable to discuss the derogatory and threatening voice content out of selfless concern for their loved ones: *‘I would rather it just scared me than scared anybody else’* (V5). V2 heard the voice of his parents‐in‐law, but felt there were no benefits to sharing this with his wife: *‘I don’t mean to keep secrets, it’s not secrets it’s just I don’t want her to get hurt by it. [..] there’s no good can come from it by telling her.’*. He described his wife as *‘devastated’* after a previous discussion. V6 did not want family concerned about him: *‘I think I might worry them a bit too much [..] that I was ill’* and V8 thought that her family would come to harm if she told them: *‘if I told someone else they would also know, and then the [voices] would try and kill them as well’* (V8).

Participants were also mindful that voice hearing impacts the dynamics of their relationships, *‘imagine there are two pillars supporting the roof and I sort of broke down and he has to be even stronger’* (V4), and V2 was aware of the impact of hearing voices on his young son after the voices told him *‘we are going to take him off you’*, he explained *‘I couldn’t handle it [..] I had to [..] say look can we take him back to his mum tonight because I just, you know it’s not fair me being like this’*. V6 who had found it helpful to check voice content with friends was concerned that this would reduce their perception of his trust in them *‘I have insulted them a little bit[..] like I don’t trust them’*.

#### People don’t understand what I’m going through

The majority of participants felt that people around them do not understand voice hearing. For some this was due to people’s lack of experience: *‘it’s difficult for anyone who hasn’t been through it [..] to understand.’* (V2). In some instances, the lack of understanding led to people being perceived as dismissive of the participants’ experiences: *‘she doesn’t really understand. I think she has always been one to say that I should just get on and kind of you know forget about [the voices]’* (V1), and lacking empathy: *‘They all say it’s completely in my head and there’s no reality attached to it whatsoever, [..] no one really understands it’* (V15). V2, who invited his parents to listen out to the voices *‘to see if it was there or not’* explained *‘that was the frustrating part [..] they would say you know within three seconds we can’t hear nothing’* but added *‘As I probably wouldn’t if I hadn’t been through it’*. V4 explained that her family would not listen to her experiences *‘we just ended up in an argument. They don’t want to really listen [..] just say it’s illness, take medication’*. A lack of understanding resulted in some loved ones being less patient when in social situations *‘I am not being rude and not listening to them it’s just like I’m petrified listening to something else [..]. They don’t understand that do they?’* (V7). This lack of understanding also extended to mental health services: *‘I still clam up because I don’t really want to tell anyone [what the voices say..] it goes back to CAMHS [..] when I tried to tell them, I wasn’t believed.’* (V5).

#### We don’t share the same reality

Hearing DTVs reduced the common ground with other people: *‘because of the kind of er more of a focus on trying to rid myself of the noise it’s like socially you know I have very little to say in a [..] social situation.’* (V1) and because voices were not a shared experience he described *‘like I’m living two different lives at once, I have this kind of strange world in my mind and then I have the kind of physical reality of this world.’* (V1). For others, this led to loneliness: *‘Quite, quite sad[..] Because I was the only one who was hearing voices.’* (V10). V13 described a sense of disconnect from friends because their lives had not been impacted on by voices: *‘they have all got families of their own now and grown up, got families of their own, got careers and stuff. I just feel like I got left behind sort of thing.’*.

#### I fear stigma or negative judgement from others

Several participants described a difficulty talking to others because of a fear of judgement from others *‘I haven’t told anyone you know even, even best friend I would never tell them in this much detail. [..]it’s you know it sounds mad isn’t it, which it is you know’* (V2). Particular negative judgements that were feared from other people included the following: *‘he is either a pervert, peeping tom or whatever’* (V2), *‘either think you are lying [..] does she want attention, um is there something really wrong, [..] does she need to be put anywhere [into hospital]’* (V9) and *‘I would be worried in case they take the mick or something.’* (V13). For some participants, fear of judgement was triggered by voice content: *‘it’s always been [the voices saying] that everybody else hates you and they don’t need you and they think the worst of you’* (V5).

#### I might respond to the voices when I’m around other people

Responses to voices in some cases reduced the quality of relationships because *‘sadly um they can make you um not so nice to be around.’* (V7) by being *‘not aggressive but loud and [..] making people feel uncomfortable’* (V7). V4 had noticed the impact on her husband *‘there are times I have to shout back to the voices and it does make things depressing for him’*. This also affected others *‘I felt like I had not only embarrassed myself, but I had embarrassed [my family] as well’* (V7), resulted in apologies *‘I had to like say sorry like blurting it out because I heard the voice’* (V13) and led to avoidance *‘the bad results have come off the back of the voices happening. So, you just tend not to want to embarrass yourself as much again’* (V14).

#### The voices are listening to my conversation and there may be consequences from what I say

A few participants described purposefully not discussing things for fear of the voices listening *‘that’s why I stop myself at certain points and I have to go no, [..] I’m not going to talk about that’* (V15) or saying things that they hear *‘I’m having two conversations yeah, sometimes I’m talking to you and I’m saying things that I know they are listening to’* (V15). Sharing voice content was difficult for many participants, and V5 explained that for her this was *‘because I know I’m going to get a backlash. [..] I’m not allowed to tell people, that’s been drummed into me since I was a kid.’*

#### It’s confusing, working out who is speaking

A few participants described hearing voices when other people are talking: *‘sometimes like I hear my name um being called [..] but sometimes somebody is actually calling me and it’s like am I thinking or hearing it?’* (V7). V7 described this as *‘amusing’*, however, V10 found it *‘confusing because I don’t know who is talking’*. and V5 described arguments with family about what they have said (or not).

#### The concentration required is hard or tiring

Difficulty concentrating on conversations was a common experience: *‘when people are talking to me I might find it hard to concentrate on them if I am hearing things’* (V7) and the division of attention was tiring: *‘doing this plus having ten other conversations [..] it’s not taxing it’s extremely tiring’* (V2). This impacted on the ability to chat to people: *‘lost how to communicate I suppose verbally’* (V6), attend therapy: *‘after ten minutes I was just so exhausted I said look I can’t talk anymore’*. (V2) and was noticeable to others: *‘[my husband] will notice sometimes [..] we could be having a conversation and I will quickly sort of flick and [..] for that split second you are kind of distracted.’* (V9).

#### Being around people can make DTVs worse

A few participants noted that people can trigger emotion which leads to voices: *‘I don’t like talking to my mum about [the voices] because she gets upset [..], that makes me start getting upset and then the voices just get worse’* (V10). People could also serve as a direct trigger: *‘[the voices] can possess people around me and, and they can start talking through these people. And it’s like they are talking at me directly’*. (V12). Paranoia, initiated by being around people, was also a common source for voice content: *‘there was this elderly woman and elderly man[..] I think they were agents. She started talking derogatory, cynical, making fun of me, bullying me. That was a real woman, [..] one of the agents’* (V4). Being around others could also make the threatening voice content more challenging to dismiss: *‘When I am in situations when I’m around people that’s where I struggle with it feeling so real. [..] I will try and work out who is saying it, why they are saying it, [..] I guess that’s where the paranoia crosses into things’* (V5).

### The relationship between social connection and DTVs

#### Disengaging from social contact is a common initial response to hearing DTVs

Almost all participants referred to disengaging from social contact in response to hearing DTVs. This was described as 1. Isolating oneself from people *‘You know you withdraw, you don’t want to talk to people’* (V2); 2. Turning away offered support *‘friends would come to see me [..] I was just turning them away’* (V7); 3. Difficulty talking to other people *‘I would go and see my family, but I wouldn’t say anything to them really’* (V6); and 4. Difficulty sharing the experience of hearing voices *‘six months I waited before I told my mum and dad. I had already gone to the doctors at that point [..] I was just so frightened to tell anybody’*. (V9). One participant who had heard voices for 15 years had not spoken to any family or friends about hearing them.

V3 described that withdrawal came prior to the onset of his voices, initially as a consequence of depression and anxiety, but triggered his first episode of voices: *‘That was sort of I think the first that I experienced the voices. So, I think what sort of triggered it was becoming increasingly isolated and isolated from social exposure’*. (V3).

#### Social isolation can be a time of vulnerability to DTVs

Whilst there were clear reasons for isolating oneself, many participants also noted that DTVs more commonly occurred when they were alone: *‘I think it occurs if I’m by myself [..] in my bedroom, that’s when it will come’* (V11) and *‘I tend to hear them when I’m on my, mostly on my own sort of thing’*. (V13). When alone DTVs were more central in attention *‘when I am kind of on my own [..] they kind of really do occupy my kind of attention.’* (V1) and some participants were more inclined to believe what they say *‘you could beat any of the strongest people down by you know making them solitary and then keep telling them something until they believed it’* (V2). V6 summarized the following: *‘Not talking to people just made myself worse’*.

#### The experience of reconnecting and its association with recovery

Whilst some participants were still very isolated: *‘the only people that I’m really in contact with on a kind of regular basis, is my mum and her husband. I don’t really speak to him much, I speak to my mum occasionally’* (V1), around half of the participants described an increase in their social connections over time *‘I had been inside for a month [..] I was getting all these voices, but I didn’t really talk about it [..] Because the gym is [..] it’s a 100 m walk. So, I thought I would just try and, try and do it’* (V3). Some described beginning to accept offers of help *‘I have accepted the help of other people I guess. Its only then that I kind of feel like I have had some kind of a life’* (V5) and talking to people more: *‘I have been talking to people and I haven’t been shut away in my room’* (V10). These participants also tended to be the participants who were less troubled by their voices than previously. However, even those participants who had greater social recovery still chose not to share some voice content: *‘they ask me what my voices are saying I still clam up because I don’t really want to tell anyone’*. (V5).

Several participants noted an association between social connection and an improvement in their management of voices, for example: *‘the more I could open up, the more I let my mates know, the more everything has settled down really’* (V5), *‘over time talking about it helped so much. Because I found the more I supressed it the worse it got. [..]’* (V9), and *‘a lot of where I have got to now is by talking’* (V7). V3 described intentionally getting a job which involved interacting with other people as a means of managing the voices: *‘that’s sort of why I got the job, to sort of force myself to go outside and um engage with the world [..] that’s the way that I try to manage the voices is by um getting myself outside.’*.

These participants also looked back with the benefit of hindsight. V5 now speaks to friends to check out whether voice content is real or not and explained: *‘I wish that’s something that somebody had maybe said it’s alright to do that’*. V7’s top piece of advice to others was to *‘talk about it,’*, he reflected *‘the best thing I ever did was talked about it but then the worst thing I did was when I was 16 I didn’t tell anyone. And then I ended up like having a complete breakdown’*. V3 explained *‘I can’t just um stay at home all the time because I know that they will get worse again.’*.

#### Increasing social connection can have benefits for DTVs

For some participants spending any time with others was still a challenge. But several participants reflected on the positive changes to voices when they were successfully able to manage connecting with other people. These included people providing an opportunity to dispute voice content *‘[the voices] are telling me a red van is going to [..] pull me in and drive me off [..] if I voice it and then my mate can be like it’s just your voices babe, like look around there’s no red van’* (V5) but also whether the voices themselves are real or not: *‘I tell friends about it and they say I don’t hear anything you know there’s nothing there’* (V6). Socializing also moved DTVs into the background of attention: *‘I’m focused on the person [..] without even thinking about it I’m just not hearing the voices as much’* (V3). V6 noticed that socializing *‘tends to help them not, not to occur in the first place’*.

### Factors which enabled voice hearers to connect with others

Around half of participants discussed improvements in social connection over time. Below are the factors which facilitated social connections.

#### Learning to rebuild trust

When voices reduce trust in other people and trigger paranoia, reconnecting requires a process of rebuilding trust, particularly with clinicians: *‘when you don’t trust anybody there’s nothing that a stranger could help you with initially until you have built up trust and built up a relationship’*. (V2). This process takes time *‘that’s really important that you take the time to let the person trust you’*. (V5) and knowledge about that person *‘I suppose just the more you get to know someone I suppose’* (V7). Trusting also involves picking the right people to take that risk with: *‘I feel like I have got the right people around me now to be able to trust in doing that’*. but also that *‘you have got to have the right support network around you to do that’*. (V5). Trust was not a static process, but rather one that changed even on a daily basis: *‘I’m only going to let one particular friend in today because she is the one I trust today’* (V5).

#### Connecting with other people who hear voices

Several participants noted that meeting people with lived experience of hearing voices (e.g., through voluntary sector organizations) was valued for over‐coming several barriers to interacting with people, for example, for understanding *‘It just helps quite a lot [..] I can talk quite freely and open, openly about things and they are not shocked or they understand’* (V13), having a shared reality *‘it would be common ground’* (V2) and for not upsetting family and friends *‘Friends and family, they are emotionally affected by your depressive stuff, and I think in the Hearing Voices group people tend to believe you which people may not get from elsewhere’*. (V4).

#### Explaining the problem

To mitigate the risk of being judged for responding to voices, V2 described providing an explanation to neighbours: *‘I said look if you hear me knocking about at night I’m really sorry I’m going through a tough time. [..] she was really lovely, and she was like thank you for coming around’* (V2). V5 felt concerned about stigma, but learnt that most people react better than expected: *‘the only time you ever hear anything about somebody with schizo anything is when something really bad happens. So, if I tell people, people are going to think that I’m going to do that or I’m going to do this. Whereas actually the more I talk about it now the more people are like, to be fair I would never have noticed [..] And that [..] makes me open up more’*. V7 highlighted the element of choice over the narrative that’s shared in order to overcome fears of judgement *‘if I meet people now and I have to explain I say I have got a bit of depression, I get anxiety, I don’t like saying I am schizophrenic because that’s like a label and they put you in a category’*. and others commented that before sharing a diagnosis it was helpful to *‘test the waters’* (V9) to gauge how people might react, or look for signs that it is safe to share.

#### If people are judgemental then you can choose to distance yourself from them

Some participants discussed the impact of stigma and misunderstanding related to diagnoses such as schizophrenia: *‘when you come across someone that freaks out about it, it throws everything then’* (V5). However, a number of participants had learnt to reduce the impact on themselves: *‘knowing that there are going to be people that find out, that aren’t going to react well to it. And accepting that as well and taking the approach of if you don’t like it don’t be around me’* (V5). V9 agreed: *‘I think in more recent years [..] I have kind of thought [..] if people don’t like it that’s their problem, not mine. If people are there and love you and accept it then they love you and accept you as well. So, [..] they have got an issue with then they are not worth your time’*.

#### Self‐acceptance

Opening up to others first required an element of self‐acceptance for some participants and over‐coming a sense of shame: *‘Whereas now I am quite open with it, I just think [..] It is not me but it’s a part of me and why should I be ashamed of it?’* (V5). This also required accepting the bad days: *‘you are allowed to feel unwell and you are allowed to have this. And just because you have these voices doesn’t make you a bad person’*. (V9). This facilitated acceptance of help from others: *‘I have accepted the illness and I have understood it [..]I have done what I have needed for me and I have accepted the help of other people’* (V5) and for V6 improved confidence enabled him to start asking his friends whether they had also heard the voices he hears. For V2, self‐acceptance occurred spontaneously *‘now [..]I can’t be bothered to not be me’* but for V5, learning about her diagnosis was important, and for V9, non‐judgemental healthcare professionals helped: *‘Just to have someone there to listen to you and not judge you and understand you’*.

#### Knowing that some days it’s OK to stay at home

Whilst participants shared many benefits from being around other people, there was acknowledgement that on some days staying at home is more helpful, particularly when paranoia is high and this might trigger voices *‘if it’s a bad voice day and I can’t cope with feeling safe outside I just take a day to stay inside and just watch some films’* (V5) and *‘I’m in an open wide space and I’m not used, [..] to it [..] when I get like that I am used to being in my room where I, I feel safe and I can question it’*. (V10). V5 explained that this can help overcome paranoia triggered by voices: *‘when I’m by myself at least I can check everywhere in here and if there’s no one in here I can put it down to my illness. I can trust my eyes.’*.

#### Other people persevering in their efforts to provide support

The persistence of others was valued in over‐coming the instinct to disengage from people *‘you don’t want to talk to anyone and they kept ringing, they kept you know battling through until I spoke to them’* (V2). For V2, this helped because *‘it shows they care’*, and for V5, it enabled her to question the voice *‘I think they have a hard job doing it because when I believe it it’s hard to tell me otherwise. But [..] if they carry on for long enough I will calm’*. And V7 felt emotionally affected by the perseverance: *‘how loyal were my friends because they kept coming back [..] I was quite touched by that’*.

#### Shifting attention from the voices to the person

Some participants described training themselves to be less distracted by voices during a conversation. For some, this was an active process *‘I like to think I have learned how to communicate better um and make sure instead of being so inward sort of focus on the other person you know’*. (V7) and *‘if there were voices then that’s how you learn to channel them out by speaking to you and actually listening to you’*. (V2), but for others, it came naturally ‘*A lot easier than you realise’* (V12). This was described as *‘A skill that you didn’t have before’* (V2).

## Discussion

Social support is crucial though recovery from any serious illness. Yet for the participants in this study who experienced DTVs, it was difficult both to be around people and to share the experience of hearing voices, particularly at the early stages of illness. This reduces the opportunity for social support, and at first onset may lengthen the duration of untreated psychosis. Whilst the initial instinct was to disengage from people, isolation was also described as a time of vulnerability for hearing DTVs. Some participants learnt over time that being around people offered an opportunity for distraction and disputing voice content. However, this needed to be carefully managed, particularly avoiding interpersonal situations that might trigger voices (e.g., situations which trigger paranoia or strong emotion).

‘Asociality’ has previously been conceptualized as a negative symptom of schizophrenia and defined as a lack of interest in forming relationships (Marder & Galderisi, 2017). Rather than an inherent lack of interest, participants in this study described significant barriers to spending time with others, including difficulties during conversations (e.g., difficulties concentrating and working out who is speaking), voices triggering avoidance (e.g., fear of responding to voices in public, or social interactions triggering voices), and difficulties sharing experiences of voice hearing (e.g., not wanting to upset others and a lack of understanding from others). The difficulty being around people was therefore not driven by a lack of interest, but rather an understandable attempt to avoid negative experiences linked to voice hearing. Indeed, participants were thoughtful about the impact that their voice hearing had on relationships and described feeling touched by people showing that they care. The detailed findings of this in‐depth qualitative study clearly warrant further research into the social difficulties experienced by people who hear voices.

There are several potential limitations of this study. First, social interactions by their nature involve more than one person, yet this study learns from only one side of the interpersonal dynamic and at one particular point in time. Interviewing the social network of voice hearers and clinicians would offer an additional perspective. Second, participants consented to take part in this study which involved talking in‐depth about their experience, and hence, the perspectives of people who are unable to talk about their voices are likely less heard and may alter the results. Third, recruitment was not driven by this research question, and although there was considerable detail present in the findings, data saturation on the topic was not achieved. This particularly applies to factors which enabled voice hearers to connect with others, which reflect a smaller sub‐group of participants. Further studies designed specifically to answer the research question will test the findings from this smaller group.

Whilst this manuscript was focused on being around people whilst hearing DTVs, participants were also invited to describe their relationship with their voice(s), owing to the established links between social schemata, voice, and social relationships (Birchwood *et al*., 2004). Many participants described no relationship (e.g., *‘I don’t know. I don’t think I really have a relationship with them’,* V1), which parallels results from a previous qualitative study (Chin, Hayward, & Drinnan, 2009). Others described the relationship as abusive (e.g., *‘Oh, violation, persecution, harassment.’*, V4) which is understandable given that all heard DTVs. Given that these data are not related to the experience of being around people, these findings were not included in the resultant themes. However, further research investigating social schemata (assessed via quantitative methods or alternative qualitative questions) and the impact on social networks would be valuable, particularly given the existence of relational therapies (Craig *et al*., 2018; Hayward, Jones, Bogen‐Johnston, Thomas, & Strauss, 2017).

It was strikingly sad to hear participants describe the sometimes debilitating experience of hearing DTVs, alongside descriptions of feeling unable to talk to people about it, and hence not receive the social support concomitant with a severe illness. Clearly at a societal level more work is required to correct the common misconceptions about diagnoses such as schizophrenia. This might reduce the chances of a negative reception towards such diagnoses. Increasing access to existing psychological interventions might also enable a more open dialogue about voice hearing. For example, family interventions provide a safe forum for the social network to discuss voice hearing and are already a recommended treatment (National Collaborating Centre for Mental Health, 2014). Hearing voices groups (Ruddle, Mason, & Wykes, 2011) and the hearing voices network (Longden, Read, & Dillon, 2018) enable voice hearers to meet other people with lived experience of voice hearing. Lastly, interventions already exist which enable voice hearers to tackle shame (Morrison *et al*., 2016) and social recovery (Fowler *et al*., 2018). These treatment options might be further optimized by targeting the additional factors identified in this study such as fostering trust, learning to switch attention, and developing a narrative to explain voices to people. Optimizing treatments for social connections is important in its own right. The potential to also impact on voice hearing, as described in this study, suggests that social networks might be a vehicle for disengaging from and disputing DTVs. This warrants further investigation.

## Conflicts of interest

All authors declare no conflict of interest.

## Author contribution

BS, LJ, DF, and LG conceived of the study with consultation from the McPin Hearing Voices Lived Experience Advisory Panel. BS collected the data. BS, EČ, and the McPin Hearing Voices Lived Experience Advisory Panel analysed the data with supervision from LG, DF, and LJ. BS drafted the manuscript, all authors edited it, and approved the final version for publication. BS takes overall responsibility for the integrity of the work.

## Supporting information


**Appendix S1.** Supplementary materials: supplementary methods, interview schedule and audit trail.

## Data Availability

In order to maintain the confidentiality of participants, data are not accessible.
