# Supplementary material for: The challenges and opportunities of social connection when hearing derogatory and threatening voices: A thematic analysis with patients experiencing psychosis
Source: Psychol Psychother. 2020 Oct 30;94(2):341–56. doi: 10.1111/papt.12303 (PMC8247012; doi:10.1111/papt.12303)
Supplement: Supplementary file 1 — Appendix S1. Supplementary materials: supplementary methods, interview schedule and audit trail. [file PAPT-94-341-s001.docx]

**Supplementary materials**

**Supplementary materials - contents:**

1. **Supplementary methods**
2. **Interview schedule**
3. **Audit trail of key decisions made throughout the study**
4. **Supplementary methods**

*Methods*

BS is a female clinical psychologist who has worked with people who have experience of hearing voices. EČ (second rater) is a clinical psychologist who has worked with people who have experience of voices and specialises in dissociation research. The interviewer’s interpretation of descriptions was checked within the interview using pocket summaries. The final themes, and full written results were reviewed by the lived experience advisory panel and two participants to triangulate the results.

1. **Semi-structured interview schedule. Edits were made in accordance with the emerging theory reported in (Sheaves et al., 2020).**

***Preamble:***

We’ll now move on to the interview section of the meeting. This is an opportunity to share, in your own words what it is like experiencing negative or threatening voices. People use many different words to describe these voices, like ‘abusive’, ‘negative’, ‘harmful’ – what word would like use to use to describe these voice(s)?

Ok, thank you, so we’ll describe the voice(s) as the ‘ ………….[insert participants word]’ voice(s).’

So this part of the meeting is all focused on the ….. voice(s). I’m interested in all aspects of your experience, but my key interest is what it is that makes voices so convincing and believable, and therefore makes them difficult to ignore. Whilst I have some questions here to guide us, I am interested in anything which is particularly important to your experience - your own story. We can therefore use these questions flexibly.

These sorts of interviews are different to day to day conversations in that it may feel like you are talking more and me much less. This is very usual, but if it feels uncomfortable just let me know. Equally if there are any questions which you find upsetting please do let me know, we don’t want the interview to be a difficult experience for you so we could pause, move onto a new topic or stop completely at any time.

Before starting some people find it helpful to know a bit about me. I am a clinical psychologist, which means I offer CBT (talking) therapy. I work within a research team where we aim to design CBT therapies which are more helpful for people. I am currently focused on developing more treatments for people who hear abusive voices and this is the purpose of speaking to you today, so that we can learn how we might better help people.

***Interview:***

1. I wonder if you could start by telling me your story of hearing voices. You can start wherever seems right to you.

*PROMPTS*

*-“When did the voice(s) start?”*

*-“Do you feel able to share what the voices say / said?”*

*-“What do/did the voices sound like?”*

*-“Do/did you experience visions, smells, tastes or touch related to these voices specifically?”*

*-“Do/did they have an identity?*

*-“How do you make sense of the voices?”*

*-Why do you think that you hear them?”*

*-“Where are the voices?”*

*-“Can other people hear the voices that you hear?”*

*-“Do/did you think anything triggered the voices in the past?”*

*-“Do/did they relate to your experiences?”*

*-“Did you tell anyone about hearing voices?” “what was that like?”*

*-“What was it like first making contact with mental health services?”.*

1. Identify cognitions driving engagement with voices: *“when do/did you find you listen(ed) to them?”*, “*why?”.*

*PROMPTS*

*-“Do/did you believe what the voices say when you hear them? Why / why not?”*

*-“And what about later, when they may be less intense?”*

*-“Has anything ever made you question the things that they say?”*

*-“How would they do what they say they will do?”*

*-“Have/did you ever thought about not listening to them?”*

*-If no choice but to listen – “why is that?”*

*-“How much time do/did you spend listening to them?”*

*-“Have you ever thought about trying to change their mind?” “why?” “why not?”*

*-“What have you tried?”*

*-“Have you been offered medication?* If yes: *“Did that have any effect on the voices?”*

1. The consequences of listening to voices: *“What happens/happened when you listen to the voice(s)?”.*

*PROMPTS*

*-“How do/did you feel when you listen to the voices?”*

*-At the time*

*-And afterwards*

*-“What do/did you do?”*

*-“What do/did you think about yourself / the world / other people?”*

1. Identify fluctuations in experience: *“Are/were there times when the voices are better?” “Are/were there times when the voices are particularly bad?”*
2. Elicit psychological mechanisms of change: *“Why do you think this changes/changed?”*, “*Have you ever thought about dealing with them in a different way? What was that like?”.*

*PROMPTS*

*-“How do/did you cope with the voices? …And what is that like?”*

*-For people with past experience of voices: “was there a particular turning point for you, when things became easier?”, “why do you think that was?”.*

1. Experience of therapy with voices: *-“Do/did you hear voices when talking to other people? What is/was that like?”*

*PROMPTS*

*-“What is/was your relationship like with your voice(s)?”*

*-“What do you think it would be like to have talking therapy (like CBT)?”*

*-“If difficult: Is there anything that a therapist could do or say to make it easier?”*

*-“What would you want/have wanted out of therapy?”*

*-“What would it be like if these voices were less present in your life?”*

1. Additional information about the experience of voice hearing: *“Is there anything else which is important to your experience of hearing voices which we haven’t spoken about yet?”*.

*PROMPTS*

*-“Do you have any advice for other people who hear these kinds of voices?*

*-“Any advice for health care professionals?”*

For the interviewer to check their understanding within the interview: “I might think about it like [*insert paraphrase*]… what do you think of that?” “How would you change that?”. Or, “what I’ve heard from you is [*insert paraphrase*], is that right?”

If interviewer thinks the participant might appreciate a break: “would you like to pause for a break here?”

To end: some people find it helpful to have a quick phone call after the session, just to see how they are feeling after sharing their experiences. Would you appreciate a call later today / tomorrow?

1. **Audit trail of key decisions made throughout the study**

| **Point in the research** | **Considerations** | **Outcome** |
| --- | --- | --- |
| Protocol | Original aim related to this research, as stated in the protocol:  To learn about the experience of engaging in a conversation whilst hearing an abusive voice(s) and how this may (or may not) impact on a therapeutic relationship. | Upon completion of the data collection it was clear that this question had relevance beyond just a therapeutic relationship. Hence the research question was amended to:  ‘To understand the experience of being around people whilst hearing derogatory and threatening voices.’ |
| Initial coding was completed by BS concurrently to analysis reported in Sheaves et al., (2020). | Codes from Sheaves et al. (2020) considered relevant to this study:   1. Voices and social interactions. 2. Isolation. 3. Trust. 4. Paranoia. 5. Stigma. 6. Worry what other people think. 7. Advice for other voice hearers. 8. Advice for clinicians and therapy non-specifics. 9. Reality checking. 10. Things that helped. | Quotes related to the 10 codes (listed left) were pooled and read several times. A rough coding framework was generated specifically related to the experience of being around people whilst hearing derogatory and threatening voices:   1. Isolation and reduced social networks. 2. Don’t talk to people about hearing voices. 3. Speaking to other people is hard (including 13 reasons why speaking is hard). 4. Speaking to other people is helpful (including six reasons why). 5. Speaking to people is OK (very few quotes). |
| Initial coding related to this research question | 1. Decision re: whether to keep isolation and voice hearing as two separate constructs or describe the relationship between them. | 1. The majority of quotes do not separate the two constructs. They therefore appear connected. Based on quotes the decision was made to create an over theme of: a bidirectional relationship. Plan: to propose a bi-directional vicious cycle between social isolation and severity of derogatory and threatening voices (DTVs) to other team members. |
| Quotes second rated by EČ and discussed | Key discussion points:   1. Need to outline the experience of isolation in detail. Discussed quotes which fell into the following categories: withdrawal, turning away support, difficulty talking to people, not speaking about voice hearing. 2. Discussed exceptions to the bi-directional cycle, particularly where people trigger voices and how this should be conceptualised. 3. The issue of self-acceptance was highlighted as seeming important to two people’s experience. 4. Discussion re: the impact of voices on the dynamics of relationships. 5. ‘We don’t share common ground’ was a sub-theme for reasons why it is difficult speaking to people. However this was felt not to capture the lack of ‘shared reality’ described by V1. | 1. Isolation now described in detail. 2. The exceptions did not completely fit with the framework. Hence framework discussed with supervisory team (DF & LJ) and LEAP. 3. To assess whether self-acceptance fits with other participants’ descriptions. 4. Dynamics of relationship were negatively impacted, hence this was included under ‘talking about voices might hurt or upset others’. 5. This was discussed and the consensus was to rename the code to: ‘We don’t share the same reality’ and check with the LEAP. |
| Coding framework proposed to wider academic team (DF and LJ). | Key discussion points raised:   1. DF and LJ wondered whether the relationship is best conceptualised as a bi-directional relationship between isolation and severity of DTVs, or rather whether the relationship is more complex and nuanced. 2. DF and LJ queried whether there is a better name to capture isolation given that it is not just isolation from people but also difficulties with being around people. 3. DF and LJ queried whether participants had shared experiences of re-engaging and how they had managed this. | 1. To open a discussion with the LEAP about the nature of the relationship between isolation and voice hearing. 2. To change the term isolation to low social connectedness. 3. To go back to original transcripts to search specifically for any description relating to ways in which participants had shared experiences of re-engaging with people and how they had managed this. |
| Discussion with LEAP | 1. The LEAP considered the term ‘social connectedness’ challenging to relate to particularly when negatively phrased (e.g. “low social connectedness led to …”). 2. The LEAP related very much to the sub-headings describing the reasons why speaking to people is hard. 3. The LEAP agreed that the management of social interactions is a challenge. Discussed issues of trust, taking a risk in talking to people, being invited to less events by other people. 4. The LEAP fed-back that the relationship between being around people and the severity of voice hearing was complicated and not simply a two way cycle. Being around people did not always make voice hearing better and there were instances in which they found that limiting interactions was helpful and also that people can trigger a worsening of voices. But complete or long-term isolation was agreed to be unhelpful for voice hearing. 5. ‘We don’t share the same reality’ code naming was checked. 6. Suggested looking specifically for instances where the transcripts discuss medical professionals. The LEAP shared examples where interacting with medical professionals had been a challenge. | 1. The LEAP suggested ‘social engagement’ or ‘social interactions’ or ‘socialising’. Social interactions was felt to not capture the withdrawal aspect of the quotes, and hence social engagement was chosen. 2. No actions needed. 3. Agreed it was important to create a code related to management of interactions and revisit the original transcripts to code for this. BS and the LEAP reflected on why this had not already emerged as a code and wondered if it was because some people were still struggling with interactions. The group wondered if there are two groups of participants: i) those that still find interactions a challenge and unhelpful for voice hearing and ii) those who find socialising a helpful coping strategy. 4. The LEAP and BS agreed on the following coding framework: 1: Reasons why interacting with people is difficult. 2. Why long-term isolation is not the best solution for voice hearing. 3. Ways of managing interactions. 4. The potential benefits for voice hearing. To revisit original transcripts and look intentionally for instances where people made voice hearing worse. 5. The LEAP agreed that the ‘We don’t share the same reality’ code was appropriate. Several members related to that phrasing. 6. BS agreed to create a code for ‘medical professionals’ and be alert to this when revisiting transcripts. |
| Coding transcripts with new coding framework | All transcripts were re-coded with the new coding framework. BS was particularly alert to any examples where participants had shared that i) being around people made the voices worse (coded as ‘down sides to interacting’) ii) instances where participants had discussed interacting with medical professionals and iii) self-acceptance. | Outcomes and decisions:   1. Medical professionals code included a diverse set of quotes which were double coded in other coding categories. Hence the decision was made to delete as a stand alone code. 2. The ‘management of social interactions’ was as expected based on fewer participants. To be acknowledged in the discussion. 3. The ‘potential benefits of social interactions for voice hearing’ code was related to the ‘why long-term isolation is not the best solution for voice hearing’ code. There was significant double coding, hence the codes were merged and titled ‘social interactions and the course of derogatory and threatening voices’. 4. ‘Wider benefits’ was a code that was under ‘reasons why long-term isolation is not the solution’ code. The quotes were diverse in nature, and with little details (and hence not deemed central to the individual’s story), hence this has not been included in the results. 5. Code changed name: ‘I’m fearful of the consequences of talking about the voices’ changed to: ‘The voices are listening to my conversation and there may be consequences from what I say’ because there wasn’t enough evidence from the data that it was necessarily fear based. Data used the phrase ‘being under scrutiny’ and ‘difficult’ but not necessarily feeling scared. 6. I isolate myself because social interactions can trigger negative emotions that make voices worse changed from ‘can trigger voice hearing’ because the quotes describe negative affect as a mechanism. Paranoia triggering voice hearing was later merged with this code, and instances where people directly triggered voice hearing. Hence, code renamed: ‘being around people can make derogatory and threatening voices worse’. 7. ‘Down sides to interacting’ was double coded with quotes which also fell under ‘reasons why interacting with people is difficult’. Whilst a helpful process to intentionally search for negative experiences of interacting, following the LEAP feedback, it in fact validated the fact that the quotes had already been captured under different headings and was hence deleted as a separate code. 8. ‘voices can make me difficult to be around’ has overlap with ‘I might respond to the voices’ so codes merged. 9. Social engagement within the analysis sometimes read as if it was referring to one event. Hence after consultation with DF and LJ social connection was chosen (rather than connectedness which was initially questioned by the LEAP) and agreed with the LEAP upon reviewing the manuscript. |
